# Comparison of women with high vs. low food addiction tendency: a pilot study with voxel-based morphometry

**DOI:** 10.1186/s40337-020-00288-2

**Published:** 2020-04-01

**Authors:** Anne Schienle, Isabella Unger, Albert Wabnegger

**Affiliations:** 1 Clinical Psychology, University of Graz, Universitätsplatz 2/III, A-8010 Graz, Austria; 2 BioTechMed, Graz, Austria

**Keywords:** Food addiction, Voxel-based morphometry, Inferior frontal gyrus, Disinhibited eating

## Abstract

**Background:**

The concept of ‘food addiction’ (FA) posits that highly processed food with added fat and/or refined carbohydrates is capable of triggering addictive-like eating behavior. FA may be one possible phenotype in obesity.

**Methods:**

The present voxel-based morphometry (VBM) study compared data from three groups of women. One group scored high on the Yale Food Addiction Scale (YFAS) and was overweight (*n* = 21), whereas the two other groups had low YFAS scores and were either overweight (n = 21) or normal-weight (n = 21).

**Results:**

Overweight women with high YFAS scores had less grey matter volume (GMV) in the inferior frontal gyrus (IFG) than overweight women with low FA tendency, who in turn had less GMV in the IFG than the normal-weight group. The IFG is involved in response inhibition, which is relevant for the control of appetite and food intake. In the group with high FA tendency, the frequency of binge episodes was substantially correlated with the YFAS scores, and 11 women of this group were diagnosed with binge-eating disorder (BED). The association between IFG volume and YFAS scores was not statistically significant anymore when controlling for the effect of binge frequency as revealed by partial correlation analysis.

**Conclusion:**

This VBM study revealed an association between reported FA tendency and a neural correlate of disinhibited eating. Future studies with bigger sample sizes are needed in order to demonstrate that FA is sufficiently different from existing conditions (e.g., BED) to warrant classification as a distinct disease phenotype.

## Plain English summary

Highly processed food (e.g., fast food) may be capable of triggering addictive-like eating (craving, overconsumption despite negative effects, withdrawal symptoms). ‘Food addiction’ (FA) may be one factor that contributes to excess food consumption and obesity. The present brain imaging study compared the brain structure between three groups of women: one group reported a high FA tendency and was overweight, whereas the two other groups had a low FA tendency and were either overweight or normal-weight. The group with a high FA tendency was found to have a smaller volume of a specific brain region, the inferior frontal gyrus (IFG) than the other two groups. The IFG is involved in the control of appetite and food intake, which is impaired in FA. Since FA showed a substantial overlap with a diagnosis of binge-eating disorder, the specificity of the FA concept is questionable.

## Introduction

Food environments that include advertised, easy-to-access and inexpensive high-caloric food (‘fast food’) elevate the risk for overeating and weight gain (see Bosswell & Kober [1] for a meta-analysis). Additionally, an addiction process may contribute to excess food consumption. Gearhardt et al. [2] were among the first authors who described a condition labeled ‘food addiction’ (FA). FA has been defined as addictive-like eating of highly processed food with added fat and/or refined carbohydrates [2]. The FA concept is based on the clinical criteria of substance-related and addictive disorders (SRAD) in DSM-5 [3]. These criteria include excessive and persistent consumption of a substance despite adverse effects, craving, development of tolerance and withdrawal symptoms, attempts to quit consumption of the substance, and functional impairment.

It is generally agreed upon that food is a primary reinforcer that is able to activate the brain reward system. Even the sight of food, especially food of high energy density, engages neural reward circuitry (e.g., frontostriatal regions). This response is particularly pronounced in overweight and obese individuals (e.g., [4, 5]).

Other reinforcers, such as psychoactive drugs, elicit similar neural reward responses (e.g., [6]). This has led some researchers to hypothesize a common neural basis of FA and other types of addiction (e.g., [7, 8]). Garcia-Garcia et al. [6] conducted a meta-analysis on reward processing in obesity and addiction. Both patient groups showed altered brain activation in several brain regions including prefrontal areas (e.g., inferior frontal gyrus), subcortical structures (e.g., basal ganglia) and sensory areas compared to control subjects. Additionally, individuals with obesity and substance addictions exhibited similar hyperactivity in the amygdala and striatum when processing the problematic stimuli (food and drug-related stimuli, respectively).

The Yale Food Addiction Scale (YFAS [2, 9]) is currently the only validated measure to operationalize addictive-like eating behavior. The YFAS has been translated and validated in a number of languages, including German, Chinese, Japanese, and Spanish [10–12]. High scores on the YFAS are positively associated with the body mass index (BMI) of a person (e.g., [13]). Moreover, a positive YFAS diagnosis has been linked with the presence of binge-eating disorder (e.g., [14, 15]) and bulimia nervosa [16].

A few neuroimaging studies have investigated FA. In a functional MRI study by Gearhardt et al. [17], YFAS scores were positively correlated with activation in the anterior cingulate cortex (ACC), the orbitofrontal cortex (OFC), and the amygdala, in response to the anticipated receipt of food. All aforementioned regions encode the motivational value of food cues and are part of the brain reward system [18]. In a recent study from the same research group [19], overweight and obese patients with FA were found to exhibit diminished activation in the superior frontal gyrus to minimally processed foods, whereas control participants exhibited elevated activation in this executive control region. A resting-state functional connectivity analysis by Contreras-Rodriguez et al. [20] found that symptoms of FA correlated with greater changes in ventral caudate-hippocampus coupling between fasted and fed conditions. To the best of our knowledge, there has only been one structural MRI study carried out on FA. Beyer et al. [13] investigated a large population-based sample (*n* = 625) and found that YFAS scores were not associated with gray matter volume (GMV). However, of the participants, only 56 showed three or more symptoms of FA, and only eight participants fulfilled the diagnostic criteria of FA.

The current investigation used an extreme- groups approach with three samples. We invited selected overweight women with high YFAS scores. This group was compared with overweight women with low YFAS scores. Additionally, we studied a third group: normal-weight women with low YFAS scores. It was hypothesized that women with high YFAS scores would show altered grey matter volume in central hubs of the brain reward system (e.g., increased striatal volume, decreased frontal volume).

## Method

### Sample

A total of 677 women (mean age = 24.7 years; SD = 8.6) completed an internet-based survey that included the validated German version of the Yale Food Addiction Scale (YFAS 2.0 [21];) and the Behavioral Inhibition System (BIS) and Behavioral Approach System (BAS) scales (German version by [22]). Additionally, demographic data, body weight, and height were assessed in the survey. The URL to the survey had been provided to students and employees of the university.

Based on the YFAS data of the online survey, 63 women with a mean age of 25 years (SD = 6.25) were invited to the MRI study. We selected those women with YFAS scores > 4 (high YFAS/overweight; *n* = 21) and then matched a sample with comparable BMI but low YFAS scores between 0 and 2 (low YFAS/Overweight; *n* = 21). Normal-weight women with no FA tendency (YFAS score = 0) were assigned to the third group (n = 21).

Exclusion criteria for the MRI study were reported diagnoses of depression (severe depressive symptoms), SRAD (drugs), psychotic symptoms, and neurological disorders because of the association with altered brain volume. MRI incompatible conditions (e.g., pregnancy, metal implants, tattoos in the head/neck area) also led to exclusion from the study. The majority of MRI participants were University students (85%), the remaining participants were white-collar workers (15%).

In the MRI lab, we measured the height (to the nearest millimeter using a stadiometer) and weight (to the nearest 0.1 kg using a digital scale) of each participant. The body mass index (BMI = kg/m^2^) was computed. (The reported BMI in the internet-based survey was highly correlated with the computed BMI in the lab: r = .98, *p* < .001). A person with a BMI ≥ 25 was considered overweight (a BMI < 25 was defined as normal-weight).

Written informed consent was obtained after a full explanation of the testing procedure. The study was conducted in accordance with the Declaration of Helsinki and had been approved by the ethics committee of the University.

### Procedure

The investigation was divided into three parts: the online survey, a diagnostic session, and a subsequent MRI session. During the online screening, the participants completed the YFAS 2.0 (German validated version by [21]) that assesses dysfunctional (addiction-like) eating during the past 12 months. The questionnaire consists of 35 items (e.g., ‘When I start to eat certain foods, I eventually eat much more than I had planned’). Response categories are 0 (never), 1 (less than monthly), 2 (once a month), 3 (2–3 times a month), 4 (once a week), 5 (2–3 times a week), 6 (4–6 times a week), 7 (every day). The YFAS 2.0 includes two scoring options: (a) a continuous symptom count (possible range: 0–11) and (b) a diagnosis of FA based on the number of symptoms met and the presence of clinically significant impairment or distress. Cronbach’s alpha of the YFAS 2.0 was .93 in the present sample.

The BIS/BAS scales (German version by [22]) address individual dispositional differences in the sensitivity to reward and punishment. Typical items are ‘I feel worried when I think I have done poorly at something important’ for the BIS scale and ‘When I see an opportunity for something I like I get excited right away’ for the BAS scale. Response categories range from 1 (very true) to 4 (very false). The BIS scale had a Cronbach’s alpha of .76, and the BAS scale of .80 in the present sample.

During the first session, a clinical interview was conducted by a board-certified clinical psychologist in order to screen for DSM-5 diagnoses with a focus on eating disorders. Within this context, the number of binge-eating episodes per week was assessed. During the second session (approximately 1 week later), the MRI was performed.

#### MRI recording and VBM analysis

The MRI session was conducted with a 3 T scanner (Skyra, Siemens, Erlangen, Germany) with a 32-channel head coil. Structural images were obtained using a T1-weighted MPRAGE sequence (voxel size: 0.9 × 0.9 × 0.9 mm; 192 transverse slices, FoV = 224 mm, TE = 1.88 ms, TR = 1680 ms; TI = 1000 ms, flip-angle = 8°). The structural scans were analyzed with the Computational Anatomy Toolbox (CAT12; v 12.6) implemented in SPM12 (v7487; Wellcome Trust Centre for Neuroimaging; http://www.fil.ion.ucl.ac.uk/spm/software/spm12/) in order to gain voxel-wise comparisons of GMV.

Prior to normalization, each individual image was repositioned by setting the origin manually to the AC/PC line. Structural data were segmented into grey matter, white matter and cerebrospinal fluid. Mainly default settings of the CAT12 toolbox were applied. To compensate for the effect of spatial normalization, images were modulated, as spatial normalization could lead to volume changes. This approach preserves the total amount of grey matter. The final resulting voxel size was 1.5 × 1.5 × 1.5 mm. For quality assurance, we checked resulting images for homogeneity. As all images had high correlation values (> 0.87), none of the images were discarded. Finally, grey matter images were smoothed with a Gaussian kernel with a full width at half maximum (FWHM) of 8 mm.

### Statistical analyses

For the self-report variables (BIS/ BAS scores, number of binge episodes), an analysis of variance (ANOVA) was computed in order to compare the three groups (low-YFAS/normal-weight; low-YFAS/overweight; high-YFAS/overweight). Significant effects were followed up by t-tests.

For the GMV data, an analysis of covariance (ANCOVAs) was computed in order to compare the three groups. To correct for differences in brain size, the total intracranial volume (TIV) was implemented as a covariate. Images were thresholded for all analyses with an absolute threshold of < 0.1. Based on previous neuroimaging studies on substance-bound addictions and FA [17], [19], [23–26], we selected the following regions of interest (ROIs): insula / frontal operculum (gustatory cortex), amygdala, inferior frontal gyrus (IFG), anterior cingulate cortex (ACC) and basal ganglia. We used masks with a 25% threshold derived from the Harvard–Oxford cortical structural atlas center for morphometric analysis, MGH-East, Boston/MA, USA). The masks were resliced to a voxel size of 1.5 × 1.5 × 1.5 mm with nearest-neighbor interpolation. For all analyses, results were small-volume corrected and considered significant if the peak-level statistic was below *p* < .05, corrected for family-wise error (FWE).

## Results

### Self-report data

The three groups did not differ in mean age (F(2,60) = 2.46, *p* = .094, partial eta^2^ = .08). As intended, the YFAS scores differed between the groups (F(2,60) = 122.72, *p* < .001, partial eta^2^ = .80; see Table 1). The high-YFAS group had a higher symptom count compared to the other two groups (both *p* < .001; see Table 1). In the high-YFAS group, 11 women (52.4%) were diagnosed with severe YFAS 2.0 food addiction (six or more symptoms plus clinically significant impairment/distress), and 2 (9.5%) with moderate food addiction (four or five symptoms plus impairment/distress).

**Table 1:** Characteristics of the three groups (means, standard deviations)

	low-YFAS/ normal-weight	low-YFAS/ overweight	high-YFAS/ overweight
**M (SD)**			
**Age (years)**	22.57 (2.69)^a^	22.90 (2.59)^a^	25.19 (6.18)^a^
**Body mass index**	22.34 (1.93)^a^	28.30 (3.40)^b^	29.23 (3.59)^b^
**YFAS symptom count**	0.0 (0.0)^a^	1.05 (0.86)^b^	6.57 (2.25)^c^
**BIS score**	2.99 (.46)^a^	3.09 (0.61)^a^	3.23 (.47)^a^
**BAS score**	3.15 (.34)^a^	3.16 (0.51)^a^	3.14 (.44)^a^
**Number of BE/ week**	0.17 (0.43)^a^	0.69 (0.73)^a^	1.81 (1.33)^b^

Footnote: *YFAS* Yale Food Addiction Scale, *BIS* Behavioral Inhibition System, *BAS* Behavioral Approach System¸*BE* binge-eating episodes, *FA* Food Addiction

Different superscript letters (per line) indicate statistical significance (*p* < .05)

The BMI (lab measurement) differed between groups (F(2,60) = 13.73, *p* < .001, partial eta^2^ = .31). The BMI was higher in the two overweight groups compared to the normal-weight group (F(2,60) = 13.73, p < .001, partial eta^2^ = .31; both *p* < .01). The two overweight groups did not differ from each other (*p* = .91).

The three groups differed in the number of reported binge-eating episodes per week (F(2,60) = 17.88, p < .001, partial eta^2^ = .37). The high-YFAS/overweight group reported more binge episodes than the low-YFAS/overweight group, which in turn reported more binge episodes than the low-YFAS/normal-weight group (all *p* < .007; see Table 1). In the high-YFAS/overweight group, the YFAS scores were positively correlated with the frequency of binge episodes (r = .56, *p* = .009). Due to the restricted variance of binge frequency in the other two groups (mode = 0), no correlations were computed.

The clinical psychologist diagnosed three women of the high-YFAS/overweight group with binge-eating disorder (BED; DSM-5: 307.51). Additionally, eight women of this group received the diagnosis ‘BED of low frequency’ (DSM-5: 307.59; all of the criteria for BED are met, except that the binge eating occurs, on average, less than once a week and/or for less than 3 months).

The three groups did not differ in their scores on the BIS scale (F(2,60) = 1.13, *p* = .33, partial eta^2^ = .04) and BAS scale (F(2,60) = .01, *p* = .99, partial eta^2^ = .001; see Table 1).

### VBM

**Fig. 1:**
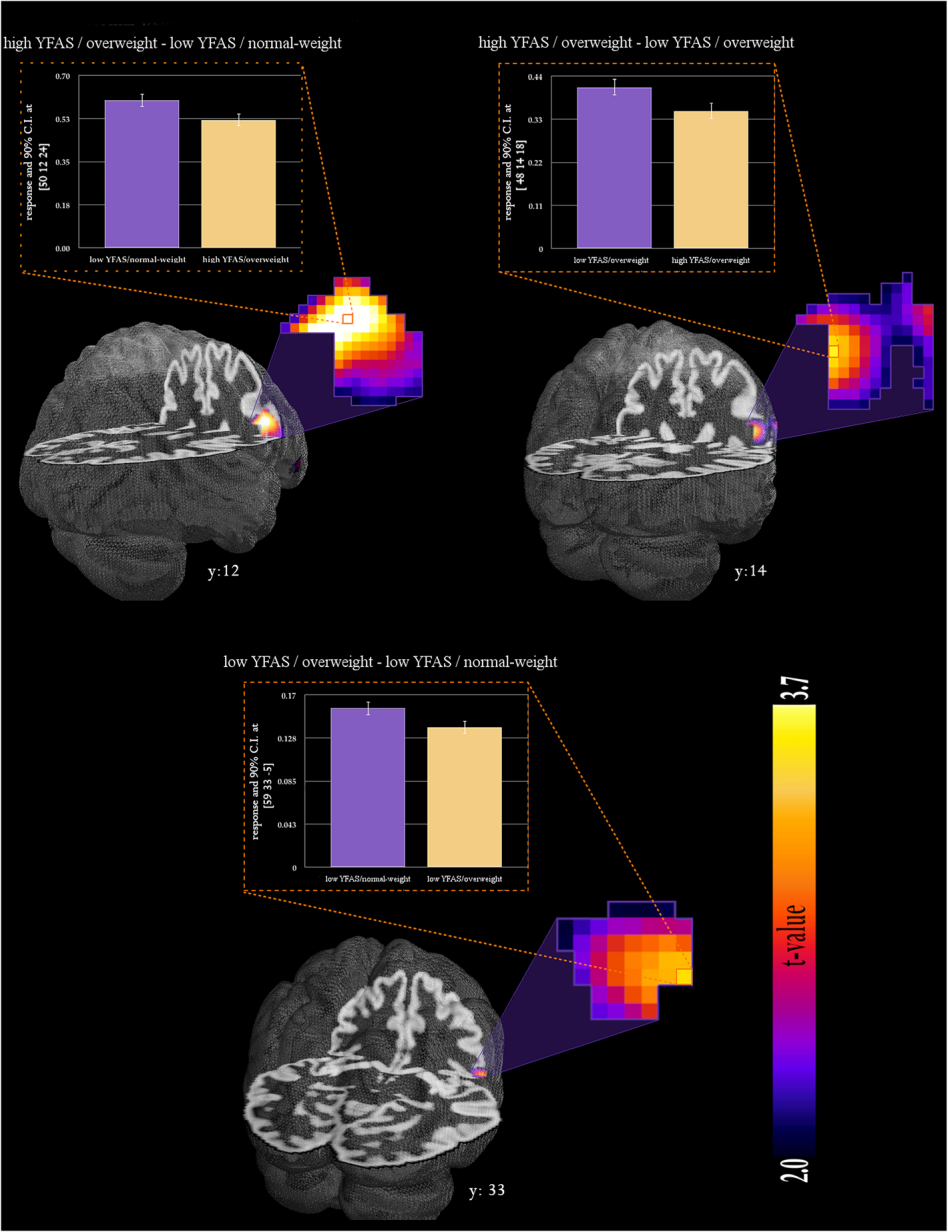
Group differences in grey matter volume of the inferior frontal gyrus

The results of the voxel-based morphometry analysis are displayed in Table 2 and Fig. 1. Women in the high-YFAS/ overweight group had less GMV in the right inferior frontal gyrus (IFG) compared to the two other groups. Moreover, the high-YFAS group displayed less GMV in the caudate nucleus than the low-YFAS/ normal-weight group and less GMV in the left IFG than the low-YFAS/ overweight group. The low-YFAS/ overweight group had less GMV in the right IFG compared to the low-YFAS/ normal-weight group.

**Table 2:** Differences in grey matter volume between groups

Region of interest	H	***x***	***y***	***z***	***t***	p(FWE)
***high YFAS/overweight*** < ***low YFAS/normal-weight***						
Inferior frontal gyrus	R	50	12	24	3.91	.0121
Caudate nucleus	L	−11	−8	17	3.85	.0319
***high YFAS/overweight*** < ***low YFAS/overweight***						
Inferior frontal gyrus	R	48	14	18	3.47	.0346
Inferior frontal gyrus	L	−57	11	27	3.79	.0175
***low YFAS/overweight < low YFAS/normal-weight***						
Inferior frontal gyrus	R	59	33	−5	3.39	.0347
***low YFAS/normal-weight*** < ***low YFAS/overweight***						
No statistically significant effects						

Foot note: *H* Hemisphere, x,y,z = MNI coordinates, t = t-value, p(FWE) = voxel-peak *p*-value corrected for family-wise error

Additionally, we conducted exploratory partial correlation analyses in the high-YFAS/overweight group to follow up on the finding of a positive association between reported binge frequency and FA tendency. The correlation between IFG volume and YFAS scores was not statistically significant anymore when controlling for the effect of binge frequency. Similarly, the correlation between IFG volume and binge frequency was not statistically significant anymore when controlling for FA tendency (YFAS scores).

## Discussion

In the present VBM study, overweight women with high YFAS scores were found to have reduced grey matter volume (GMV) in the right inferior frontal gyrus (IFG) compared to normal-weight and overweight women with low YFAS scores.

The IFG is involved in response inhibition and attention control [27]. Response inhibition can be defined as the process by which a pre-potent, routine, or dominant response is deliberately withheld or canceled. These types of processes are involved when individuals try to control their appetite and eating behavior. For example, in an fMRI study by Tuulari et al. [28], participants were instructed to passively view visual food cues, and imagine eating the foods or inhibit their craving. The inhibition condition was associated with increased activation in the IFG. Similarly, Lopez et al. [29] reported that individuals with greater IFG activity were more capable of resisting food temptations and experienced food desires to a lesser extent. Schulte et al. [19] detected reduced IFG activation in obese and overweight patients with FA during the viewing of food images, compared to household items. Moreover, the IFG plays a critical role in addiction and substance (drug) abuse. Research using structural MRI has demonstrated that GMV in the IFG is reduced in individuals with different addictions, such as heroin or cocaine (e.g., [24–26]).

Although a decreased structural size of a specific brain region does not necessarily imply decreased function, the self-report findings of the present study also pointed to reduced inhibitory control in individuals with high YFAS scores. This group reported reduced control of food intake in the form of binge-eating. This behavior is characteristic of FA, however binge-eating episodes are also the core symptom of binge-eating disorder (BED). BED (clinically relevant BED or BED of low frequency) was diagnosed in 11 participants of the high-YFAS group. Thus, there was a substantial overlap of FA diagnoses and DSM-5 diagnoses of BED. This finding is not surprising since binge-eating episodes are characterized by diminished control, which is a core feature of all addictive-like behaviors. Similar findings have been reported by Carter et al. [30], who observed that BED patients reported significantly higher FA scores compared to individuals with no history of an eating disorder (NED). In that study, 92% of the BED group met YFAS 2.0 criteria for at least mild FA compared to only 6% of the NED group. Within this context, Gearhardt et al. [31] have argued that there might be different types of reduced control of food intake. BED patients’ out-of-control eating (binge eating) occurs during discrete periods of time, whereas overeating in FA may occur over the course of a day (‘grazing’).

Interestingly, in the high YFAS/overweight group, binge frequency was substantially correlated with the YFAS scores. Additionally, the correlation between IFG volume and YFAS scores vanished when controlling for the effect of binge episodes as revealed by partial correlation analysis. These results point to the high similarity of both constructs (FA tendency and binge-eating tendency).

In conclusion, the findings of the present investigation (substantial overlap of FA/ BED diagnoses, substantial correlations between binge frequency and YFAS scores) question whether FA is a distinct disease phenotype. In line with this critical view, Davis [32] has proposed a dimensional concept of overeating, ranging from homeostatic eating to ‘passive overeating’ and ‘disinhibited eating’. FA represents the extreme end of the continuum of disinhibited eating (which includes eating in the absence of hunger, emotional eating and loss-of-control eating as criteria). If a threshold of severity is exceeded, the diagnosis of BED may be warranted [32].

It is also important to note that the concept of FA indicates an involvement of the brain reward system. However, the present study identified no increased basal ganglia volume in FA. Indeed, contrary to our hypothesis, the high-YFAS group was found to have less GMV in the basal ganglia (caudate nucleus) compared to normal-weight participants and did not report elevated reward sensitivity.

The following limitations of this study need to be considered. We only investigated women. Therefore, the findings cannot be generalized to men. Moreover, the sample size was relatively small. Studies with larger sample sizes are warranted.

## Conclusion

The present VBM study identified an association between brain volume in a region involved in inhibitory control (IFG) and YFAS scores. Future studies are needed in order to demonstrate that FA is sufficiently different from existing conditions (e.g., BED) to warrant classification as a distinct disease phenotype.

## Data Availability

The datasets generated and analyzed during the current study are available from the corresponding author on reasonable request.

## Abbreviations

ACC: Anterior cingulate cortex
BAS: Behavioral Approach System
BIS: Behavioral Inhibition System
BMI: Body mass index
FA: Food addiction
GMV: Grey matter volume
IFG: Inferior frontal gyrus
OFC: Orbitofrontal cortex
SRAD: Substance-related and addictive disorders
VBM: Voxel-based morphometry
YFAS: Yale Food Addiction Scale
